# A qualitative exploration: questioning multisource feedback in residency education

**DOI:** 10.1186/s12909-018-1270-7

**Published:** 2018-07-24

**Authors:** Brie A. Yama, Michael Hodgins, Katherine Boydell, Sarah B. Schwartz

**Affiliations:** 1 Department of Paediatrics, The University of Toronto, Toronto, ON Canada; 2 Division of Paediatric Medicine, The Hospital for Sick Children, Toronto, ON Canada; 3 Western Sydney University, Parramatta, NSW Australia; 4 Black Dog Institute, University of New South Wales, Randwick, NSW Australia

**Keywords:** Multisource feedback, 360 degree evaluations, Medical education, Interprofessionalism

## Abstract

**Background:**

Multisource feedback (MSF), involves the collection of feedback from multiple groups of assessors, including those without a traditional hierarchal responsibility to evaluate doctors. Allied healthcare professionals (AHCPs), administrative staff, peers, patients and their families may all contribute to the formative assessment of physicians. Theoretically, this feedback provides a thorough view of physician performance; however, the ability of MSF programs to consistently impact physician behavior remains in question. Therefore, the objective of this study was to explore perceptions and prerequisites to an effective MSF program in postgraduate medical education from the perspectives of both pediatric residents and AHCPs.

**Methods:**

This exploratory study was conducted in a pediatric inpatient unit prior to implementation of a MSF program. Focus groups were conducted with purposefully recruited participants from three distinct groups: junior pediatric residents, senior pediatric residents, and AHCPs. Discussions were audio recorded, transcribed verbatim and analyzed using thematic analysis.

**Results:**

Both residents and AHCPs expressed a strong interest in the concept of MSF. However, more in depth discussions identified barriers to residents’ acceptance of, and AHCPs’ provision of feedback. Roles and responsibilities, perceptions of expertise, hospital culture/interprofessionalism and power dynamics were identified as barriers to the acceptance and provision of feedback. All groups expressed interest in opportunities to engage in bi-directional feedback.

**Conclusions:**

The identified barriers and prerequisites to providing and accepting MSF suggest limits to the efficacy of the MSF process. Our findings suggest that these factors should be considered in the design and implementation of MSF programs.

## Background

The goal of multisource feedback (MSF) in medical education is to prompt self-reflection and behavior changes that translate into better patient outcomes. However, published literature has not demonstrated this, questioning the effectiveness of MSF [1–5]. Training and licensing bodies in North America and the United Kingdom have already implemented MSF programs [6–8]. The widespread trend in medical education to move to competency based education with training advancement contingent upon milestone achievement underscores the utility of an effective MSF process, which could enable the evaluation of more challenging competencies such as communication and professionalism.

Systematic reviews have found mixed results regarding the ability of feedback to result in practice change and suggest that the influence of MSF on performance is limited in quantity and quality [4, 9]. A qualitative study revealed five conditions that influence change through MSF: the provider of feedback, content, process, specificity, and congruence of feedback with other sources [3]. Further explorations into the effects of feedback content and reactions to it [3, 10, 11], coaching [12, 13], physician characteristics [1], and other variables that influence performance improvement [14], have had little practical application. Recent studies have begun to explore the relationships between residents and allied healthcare professionals (AHCPs) during inpatient ward rounds [15]. Although evidence from both the feedback and MSF literature emphasizes the source of feedback as a major factor, there has been minimal direct investigation into the nature of the relationship between feedback provider and recipient and how it may affect MSF [16, 17]. A recent study showed AHCPs are interested in providing residents with feedback on professionalism in the outpatient setting [18]. This study aimed to further the understanding of residents’ and AHCPs’ perceptions of MSF, to identify prerequisites to an effective MSF program in postgraduate medical education.

## Methods

This study involved a pediatric inpatient unit of a tertiary care hospital where MSF had not been implemented. Research ethics board approval was obtained. Three focus groups were conducted with purposefully recruited participants from distinct groups: junior (first year) pediatric residents, senior (third year) pediatric residents, and AHCPs (nurses, occupational therapists, social workers and pharmacists). Eight to twelve individuals participated in each focus group. The sole inclusion criterion was working on the inpatient pediatric unit interdisciplinary team for a minimum of one of the preceding six months. There were no exclusion criteria.

Focus groups were 60 to 90 min in length, audio recorded and transcribed. A co-investigator who was a peer colleague of the participants in each focus group facilitated the discussion (BY, SS). A semi-structured focus group guide provided a framework for discussions.

The transcripts were analyzed thematically using Braun and Clarke’s (2006) model [19]. Codes were developed and applied to the transcripts systematically. Themes were developed from the initial coding (inductive) as well as from the original aims of the research, explicated in the focus group guide (deductive). Pope (2000) notes that a deductive overview is based on predetermined objectives, yet also acknowledges that the themes will be grounded in the data that reflects the accounts of research participants [20]. The transcripts were coded in entirety by three of the investigators (BY, MH, SS). All four members of the research team discussed the coding and interpretation of the transcripts to refine codes and identify key themes which were then mapped in charts and word documents. Emergent findings were verified using reflective conversations, audit trail, thick description, and persistent observation [21]. The team approach to analysis allowed inconsistencies between the data and themes to be debated and refined.

## Results

### Initial impressions

Upon introduction to the concept of MSF, all groups *initially* identified “everyone” as potential providers of feedback. Enthusiasm was expressed, with residents anticipating professional growth and AHCPs predicting improved interdisciplinary teamwork as potential outcomes of the process. However, further discussions revealed explicit and implicit limitations and barriers to the MSF process. Our analysis revealed concerns about (i) (mis) understanding of roles and responsibilities, (ii) perceptions of expertise, (iii) hospital culture/interprofessionalism, and (iv) power dynamics as potential barriers to the acceptance and provision of feedback. These themes are described in greater detail with illustrative quotes below.

### (i) (Mis) understanding of roles and responsibilities

Residents intimated that the acceptance of feedback from AHCPs is predicated upon assessors providing feedback that a) reflects an understanding of the dynamic and competing responsibilities faced by residents and b) remains within the scope of individual AHCPs’ expertise.

“If they (AHCPs) don’t have a clear understanding of what a resident is expected to be doing…they may not be in the best position (to provide feedback)…you may almost push out some of the stuff that you think (is) not relevant because (AHCPs) don’t quite understand the role.” (Senior Resident)

AHCPs also acknowledged that their feedback was constrained by their scope of practice:

“(Feedback) wouldn’t be about their medical expertise, it would be … bedside manner, how they interact with the family, how they presented, how team members perceive them… things that are more relationship based.” (AHCP)

AHCPs acknowledged their potential lack of understanding of residents’ training and roles, but did not perceive this to be a hindrance to their ability to provide feedback.

### (ii) perceived areas of expertise

The need for feedback to reflect scope of practice is further nuanced with *residents’ perception* that feedback providers possess expertise and not merely a professional designation.

“It’s obviously context dependent…the palliative care nurses would be way better to give you feedback observing how you broke bad news than your senior cardiologist.” (Senior Resident)

The perception of expertise was grounded in the feedback provider’s de facto clinical practice, not the presumed skills attributed to their titles or credentials.

### (iii) hospital ward culture and interprofessionalism

Residents are trainees with temporally transient roles that contrast the consistent roles of AHCPs. This cultural dynamic, superimposed on the existing hierarchy, has the potential to create tensions affecting feedback.

“Every year there are new residents, meanwhile the AHCPs remain the same, so people start getting frustrated…this person doesn’t know how this ward works’.” (AHCP)

As trainees, residents are subject to continuous evaluation via the traditional model of evaluation resulting in observable shifts in behavior.

“They’re being their true self and sometimes there’s a different attitude that comes forward when the attending comes on the ward.” (AHCP)

Despite the desire for a unified multidisciplinary team, a divide between physicians and AHCPs may exist. This separation, coupled with a lack of formal procedures for providing regular feedback, represents a barrier to feedback provision.

“If you have a concern and you would like to give some feedback but you don’t have a formal opportunity to do it…if you talk to someone about it, it becomes a very big deal - if you haven’t been asked.” (AHCP)

This has an impact on feedback received by residents.

“It really depends on the situation in which you come to learn about negative feedback...the only negative feedback I got went right to the chief residents...it’s frustrating.” (Junior Resident)

### (iv) power dynamics

All groups identified multiple tensions stemming from perceived power differentials. Generally, residents perceived the balance of power to rest more on their side.

“I’m sure there are many (AHCPs) that would perhaps feel a bit uncomfortable about…providing feedback to a physician of any standing.” (Junior Resident)

This imbalance was particularly striking when residents spoke about receiving feedback from bedside or more junior nurses compared with charge or more senior nurses.

“I’m not trying to downplay the junior nurse, it’s just I would be much, much more receptive and I would probably sit down and have a proper conversation with the charge nurse.” (Junior Resident)

However, the power possessed by AHCPs was also acknowledged, particularly when nurses perceived residents as remiss in fulfilling their responsibilities.

“Now anytime I interact with a nurse I think…do I have to walk on eggshells with you or are you going to make complaints anytime I don’t do an order the second you ask me for it?” (Junior Resident)

“There’s power differentials- if I'm your boss, if I’m your colleague, if I’m working with you- there are many dynamics...why people don’t want to (provide feedback) and shy away from it.” (AHCP)

The perceived balance of power fluctuates creating barriers to providing and accepting feedback.

### Concluding impressions

Despite an initial interest in MSF, discussions demonstrated residents’ hesitation to engage with and accept it.

“I’m just not sure what the value is from getting (feedback) from other sources other than ultimately wasting your time and potentially making you feel badly or over-inflating yourself.” (Senior Resident)

Contrastingly, AHCPs arrived at an agreement regarding the importance of MSF.

“Providing feedback to residents sends a message … that (our) opinions matter.” (AHCP)

These divergent perspectives regarding the value of MSF represent significant challenges. Despite this apparent division, each group identified the value of providing group feedback with respect to team function. Residents emphasized interest in transforming feedback to a “two-way street” highlighting the value of “bi-directional feedback”. The AHCPs agreed, commenting on the ‘very unilateral’ nature of traditional MSF, emphasizing that reciprocal feedback could promote a culture where “everybody understands that it is okay to give feedback to everyone”.

## Discussion

The identified barriers and prerequisites to providing and accepting feedback suggest limits to the efficacy of MSF. The reliability of MSF may be affected by assessor groups and the competencies assessed [22]. This study contributes by discerning key factors, which impact MSF programs. A novel feature of this study is the simultaneous exploration of the recipients’ and providers’ perspectives. Inclusion of both junior and senior residents also allowed for exploration of residents’ reflections along the developmental spectrum of postgraduate residency training; however, impressive concordance was consistently noted between these two groups. The four major factors identified were knowledge of the roles and responsibilities of others, the importance of perceived expertise, hospital culture viewed through an interprofessionalism lens, and power dynamics. Ultimately, the effectiveness of feedback is determined by the providers’ capacities to communicate feedback and the recipients’ abilities to comprehend, reflect, and implement enduring behavioral changes. Therefore, the self-identification of barriers that impair this process by residents and AHCPs is significant with the potential to influence on-going interactions.

An important aspect of this study is the focus on residents and the implications for assessment in postgraduate training. Residency differs from independent clinical practice. The intensity and rate of professional growth, combined with the complexity of interprofessional relationships during residency may impact the MSF process. Despite the minimal literature specific to the residents, programs across North America have implemented MSF with limited success in the achievement of desired outcomes and revelations regarding possible negative impacts [23]. Educators are left pondering how best to implement MSF, to achieve the intended outcomes and minimize complications. Competency based education further highlights the need for measures to evaluate competencies including communication and professionalism. This exploration of residents’ and AHCPs’ impressions of the MSF process provides insight into issues that could be directly addressed in order to optimize MSF during residency.

Residents identified AHCPs’ lack of knowledge of residents’ responsibilities as an impediment to feedback acceptance. This theme was strongest during discussions related to overnight duties when residents’ responsibilities increase and diversify. These concerns were validated by AHCPs who acknowledged that they did not understand residents’ schedules or the scope of their responsibilities. If AHCPs are unaware of the larger context within which their feedback will be interpreted, their ability to effectively communicate its relative importance will be limited. While specific solutions to this challenge should incorporate an understanding of individual institutions and residency programs, MSF provided by AHCPs must reflect an understanding of residents’ responsibilities to optimize feedback uptake by resident recipients.

Residents emphasized the value of feedback from individuals they perceive to be experts. Expertise has previously been identified as an important feature in successful feedback exchanges [3]. This study further illustrates that the perception of expertise is not limited to specific professions. While the senior physician is often the ‘expert’, residents identified AHCPs as more valued sources of feedback in certain scenarios. AHCPs supported this and further identified their expertise in the assessment of residents’ communication skills. The expansion of the residents’ view of expertise is an important finding that has implications for determining experts to assess specific competencies. As communication is one of the more challenging competencies to evaluate, residents’ receptivity to receiving feedback on this skill from AHCPs would be critical to successful integration of MSF.

Academic inpatient wards engender unique cultures with complex interprofessional dynamics. The rotation-based nature of residency contrasts the stability of AHCP positions, creating different experiences beyond those expected due to differing roles. Furthermore, residency remains a period of training during which residents are expected to identify as learners under the constant evaluation and graded supervision. While the culture of medicine has evolved over time and efforts to promote collaborative multi-disciplinary teams exist across most centers, a division between AHCPs and physicians persists [24]. The results from this study reaffirm this reality. Learning to navigate this divide and assume the identity of ‘the physician’ is an inherent rite of passage in residency [25]. This background contributes to the barriers that impede the MSF process. Our findings suggest that both residents and AHCPs recognize the impact of environment on feedback. AHCPs expressed a hesitancy to provide feedback particularly surrounding issues deemed to be ‘minor’. Residents revealed collective frustration regarding the escalation of feedback provided directly to superiors. Both AHCPs and residents acknowledged that a breakdown in the professional relationship was a frequent consequence. Information gathered informally from pediatric residents and program directors across North America confirmed that MSF can negatively impact relationships and may result in increasingly guarded interactions [23]. The culture of interprofessionalism has significant bearing on the success of MSF and any feedback process must take this into consideration.

Of all the themes identified as potential barriers to the MSF process, the shifting power dynamics may be the most challenging. There may also be a possible misunderstanding by all parties of the formative nature of feedback. While power itself need not have negative implications, it nonetheless appeared to, in the examples discussed above. As each scenario culminated in punitive consequences for involved individuals, the negative connotation was amplified. This has the potential to undermine trust in the entire process. MSF programs will need to address power differentials and provide an environment wherein both provider and acceptor could view feedback as transformative as opposed to punitive.

This study is potentially limited by design decisions. It is a nonrandom, convenience sample of self-motivated pediatric residents and staff from a single institution study. As participation was self-motivated it is possible that those who elected to participate in this study were interested in feedback to an extent that their views might not be generalizable. As with all studies which address potentially sensitive topics, participants’ responses may reflect social desirability. While this was reflected in the initial remarks about MSF, the evolution of the discussions in each focus group suggests this did not have a significant impact.

## Conclusions

The original intent of MSF in this setting, to provide resident physicians with feedback on the humanistic and relational competencies of medical practice, remains relevant despite the barriers identified in this study. The evolution of MSF to focus on team dynamics with the incorporation of bi-directional exchanges and opportunities for dialogue may overcome the barriers identified. This process could re-focus residents and AHCPs on the fundamental goal of feedback in medical education, improving skills and patient care outcomes. The next phase of this project will be to implement and evaluate such a program in the institution where this study was carried out.

Despite widespread uptake, the current MSF models have limitations that impair the desired outcomes of feedback. This study specifically identifies concerns about (mis)understanding of roles and responsibilities, perceptions of expertise, hospital culture/interprofessionalism and power dynamics as barriers to the acceptance and provision of MSF. Modification to the current MSF process as a response to the themes identified in this study should be considered in the design and implementation of future MSF programs.

## Abbreviations

AHCPs: Allied healthcare providers
MSF: Multisource feedback
